# Greed of Gold

**Published:** 1880-09

**Authors:** 


### GREED OF GOLD.

"THERE was an old man," says an Eastern parable, "who had abundance of gold; the sound of it was pleasant to his ears, and his eye delighted in its brightness. By day he thought of gold, and his dreams were of gold by night. His hands were full of gold, and he rejoiced in the multitude of his chests; but he was faint from hunger, and his trembling limbs shivered beneath his rags. No kind hand ministered to him, nor cheerful voices made music in his home. And there came a child to him, and said: 'Father, I have found a secret. We are rich. You shall not be hungry and miserable any more. Gold will buy all things.' Then the old man was wroth, and said: 'Would you take from me my gold?'"

The soul-destroying greed of gold is not a crime confined to the rich, to those who count their hoards by tens of thousands. The penniless idler may sell his soul for a dollar; many a wretch has committed murder for a less sum. Some will read the parable quoted, with a feeling of impatience and indignation, that joy and comfort and health and life should be all sacrificed in the passion of accumulating money. But it is the miser's pleasure; it is his meat and his drink; it is to him the sweetest satisfaction on earth, and he indulges in it. To hinder that indulgence, would be to ruin his body, or to blast his mind; the grave or the asylum would soon have another occupant.

But the loss of body and heart and soul together, by the destructive indulgence of any passion, or any appetite, is also a crime; and yet, in that indulgence, there may be a sweetness to the reveller passing computation. The habitual dram drinker, or opium eater, or tobacco user, or gourmand, or "*roue*," so have their habits fixed upon them, that they would pour out the miser's gold as freely as water, to gratify their craving appetites—to these, would sacrifice all they possessed; and we who are not slaves to these lusts, stand by and wonder at the infatuation. The glutton pronounces the miser a fool, and the miser pronounces the drunkard a beast. But, to all, their favorite indulgences are sweet as "honey and the honey-comb," and to break them away from them, is like taking away their life's blood—more, it is literal death.

But just as sweet, and purer far, higher and more noble, is it to cultivate and cherish and feed upon the acquisition of truth, the practice of benevolence, the promotion of our holy religion, when once the heart is in it. And as no one is a greater adept in devising ways and means for increasing his treasure, than the inveterate miser, because his whole soul is absorbed in his work, so will the searcher for truth, and the worker for its dissemination—he who live for humanity —be able to find out new truths, new ways of expressing them, new modes of distribution. Newton loved his studies, literally, more than his daily food. Multitudes of men are there, who so much love study, that the call to dinner is often regarded as an unpleasant interruption, and the meal is taken mechanically, while the soul is feeding at another table. The great Howard, and the greater—because far wider reaching—Dorothea Dix, loved benevolences so well, that the sacrifices of bodily comfort and bodily ease, for long years in succession, were counted as nothing, compared with the consciousness of "spending, and being spent" in the service of the unfortunates of their kind.

